# Socket Sealing Using Free Gingival Grafts: A Randomized Controlled Trial

**DOI:** 10.3390/dj13010024

**Published:** 2025-01-07

**Authors:** Ralitsa Yotsova

**Affiliations:** Department of Oral Surgery, Faculty of Dental Medicine, Medical University of Varna, 9002 Varna, Bulgaria; ralitsa.yotsova@mu-varna.bg

**Keywords:** post-extraction resorption, socket preservation, socket sealing, free gingival graft

## Abstract

**Background:** Post-extraction ridge resorption is an inevitable phenomenon that cannot be eliminated but is significantly reduced using additional surgical techniques known as socket preservation. They aim to create favorable conditions for implant placement and prosthetic restoration. This study aims to assess the effect of socket sealing (SS) with free gingival grafts on the vertical resorption of socket walls at the premolar and molar regions over 3 months. **Methods:** This randomized two-arm controlled trial with parallel groups (1:1 allocation) was conducted at the Department of Oral Surgery, Medical University-Varna, Bulgaria, from 27 June 2022 to 20 April 2023. Forty patients aged 30–65 were equally and randomly allocated to the SS or the control groups. Atraumatic tooth extraction was performed. In the control group, the socket was left on secondary wound healing. In the SS group, the socket orifice was “sealed” with an FGG harvested from the hard palate or maxillary tuberosity. **Results:** Data analysis demonstrated that SS with an FGG is a successful method for reducing the post-extraction resorption of the socket walls. In addition, this study confirms that the thickness of the buccal wall is a significant factor in its vertical resorption. **Conclusions:** Socket sealing with an FGG is a valuable method that eliminates the need for flap reflection and compensates for the soft tissue deficit when immediate implant placement or bone augmentation is required. Further research is necessary to determine the role of different factors influencing bone resorption and compare the effect of different socket preservation methods.

## 1. Introduction

Post-extraction ridge resorption is an inevitable phenomenon that cannot be eliminated but is significantly reduced using additional surgical techniques known as socket preservation (SP). They aim to create favorable conditions for implant placement and prosthetic restoration.

Alveolar ridge resorption is a physiological process of bone remodeling which begins during the first two weeks following tooth extraction and is associated with the disappearance of the tooth-dependent bundle bone, lining the inner surfaces of the socket, and proceeds with afunctional bone atrophy, which is a lifelong process [1]. Post-extraction resorption has been widely discussed and evaluated in the scientific literature as it can cause serious functional and esthetic challenges for subsequent dental rehabilitation [2].

The alveolar crest undergoes vertical and horizontal resorption with a vertical reduction of 11–22% of the buccal wall after 6 months. The buccal horizontal reduction is even more pronounced—29–63% after 6–7 months. After that, a reduction in the bone contour of 0.5–1% per year can be expected [3].

Post-extraction socket healing is usually secondary. The formed coagulum serves as a scaffold for the subsequent granulation tissue and bone formation. Coverage of the post-extraction areas with keratinized epithelium is established after 24–35 days [4]. However, secondary healing causes hard and soft tissue loss. Full tissue preservation is aimed especially at areas with thin periodontal biotypes and high smile lines. To date, no technique has provided such results. Therefore, additional hard and soft tissue augmentation is required in the esthetic area, both during implantation and at a later stage [5].

Recently, the focus of implantology treatment has shifted to efforts for optimal aesthetics, rather than just satisfactory functional results. Tooth extraction is followed by bone resorption and soft tissue loss, which can cause gingival recessions around the subsequently placed implants [6]. This problem can be resolved using socket sealing methods. Socket sealing can preserve the volume and shape of the surrounding soft tissues. This is achieved by covering the socket with an autogenous soft tissue graft or a barrier membrane. The technique is mainly used in the aesthetic area [7]. Another option is guided bone regeneration (GBR) during implant placement using different barrier membranes and bone substitute materials [8,9].

Primary wound closure is mandatory for socket preservation combined with GBR. Soft-tissue grafts are a reliable method, which allows for the preservation of the mucogingival junction and increases the volume of the buccal soft tissues and marginal gingiva.

Free gingival grafts were first introduced for socket sealing by Landsberg et al. in 1994. They aimed to protect the bone-grafted socket from bacterial contamination [10]. The most common donor sites are the hard palate and the maxillary tuberosity. The latter allows for thicker graft harvesting. Another advantage is the easier healing process with less postoperative pain [11]. Another key factor for graft survival is its rapid transfer to the recipient site. The inappropriate choice of suture material and technique is also a risk factor for graft necrosis. Rapid revascularization and minimal tissue reaction are expected when a microsurgical technique with thin (6/0 or 7/0) nonabsorbable monofilament suture material is used [12].

This study aims to assess the effect of SS with free gingival grafts on the vertical resorption of socket walls at the premolar and molar regions over 3 months.

## 2. Materials and Methods

This parallel two-arm randomized controlled trial with 1:1 allocation was conducted at the Department of Oral Surgery at the Medical University-Varna, Bulgaria, from 27 June 2022 to 20 April 2023 after ethical approval (118/23.06.22) from the University Research Ethics Committee and in accordance with the Declaration of Helsinki. The trial was registered on ClinicalTrials.gov (registration protocol—NCT06621498). Written informed consent was obtained from all participants before the surgery. The research was conducted in accordance with the CONSORT Reporting Guidelines [13]. The principal investigator only enrolled patients who met the inclusion criteria, while their allocation to the experimental and control groups and the sequence were randomly performed by a third party to remove bias.

### 2.1. Patient Recruitment

Forty patients aged 30–65 were enrolled by the principal investigator (R.Y.) and randomly allocated to the SS or the control groups. The participants were recruited after a detailed evaluation of the indications and contraindications for tooth extraction and SS. The patients were informed about all the advantages and disadvantages of the surgical techniques and possible complications associated with the surgical sites (including the need for a donor site in the experimental group).

### 2.2. Inclusion Criteria

1. Age between 18 and 65 years;
2. Good general health (codes Z00, Z00.00 according to the International Classification of Diseases, Tenth Revision, Clinical Modification (ICD-10-CM)—available online: https://icd10cmtool.cdc.gov/ (accessed on 12 October 2024));
3. Premolar/molar tooth requiring extraction;
4. Motivation for participation in the study.

Instructions for strict oral hygiene and smoking restrictions were given. Unwillingness to follow these instructions (e.g., refusal of smoking cessation during the perioperative period) were regarded as a lack of motivation for participation in the study.

### 2.3. Exclusion Criteria

1. General contraindications for oral surgery procedures, including but not limited to uncontrolled hypertension, diabetes, or other metabolic disorders; cerebrovascular accident in the last 6 months; recent chemotherapy and/or radiotherapy; undergoing hemodialysis; immunosuppressive treatment; recent or previous antiresorptive or antiangiogenic therapy; acute infection; pregnancy;
2. Local contraindications, such as extensive vertical bone loss before the extraction, acute local inflammation, or malignancy;
3. Patients refusing to sign the informed consent and/or lack motivation for participation in the research.

The sample size was calculated using a sample size formula for testing a hypothesis in clinical trials/interventional studies [14]:

Sample size = 2SD^2^(Z_α/2_ + Z_β_)^2^/d^2^

SD—standard deviation; Zα/2—standard normal variate for a level of significance; Zβ—standard normal variate for power; d—effect size.

Z values were taken from the Z table at type I error (5%) and 80% power (Zα/2 = 1.96; Zβ = 0.84). The SD value was taken from previously reported post-extraction vertical resorption [15]. The value of 0.8 was determined as a large effect size (d = 0.8).

Sample size = 2(0.77)^2^(1.96 + 0.84)^2^/0.8^2^ = 14.5

Simple randomization (lottery method) was selected for this trial. Patients’ initials and the last 4 numbers of their identification numbers were written on sheets, folded several times, and put in envelopes (first pile). The words “socket sealing” and “control” were written on sheets (20 times each), folded several times, and put in envelopes (second pile). A third party (a statistician who had no direct contact with the patients) picked an envelope from the first pile to identify the first patient and an envelope from the second pile to identify the type of procedure: tooth extraction with or without SS. Thus, the randomly allocated sequences and the types of intervention were determined.

**Figure 1:**
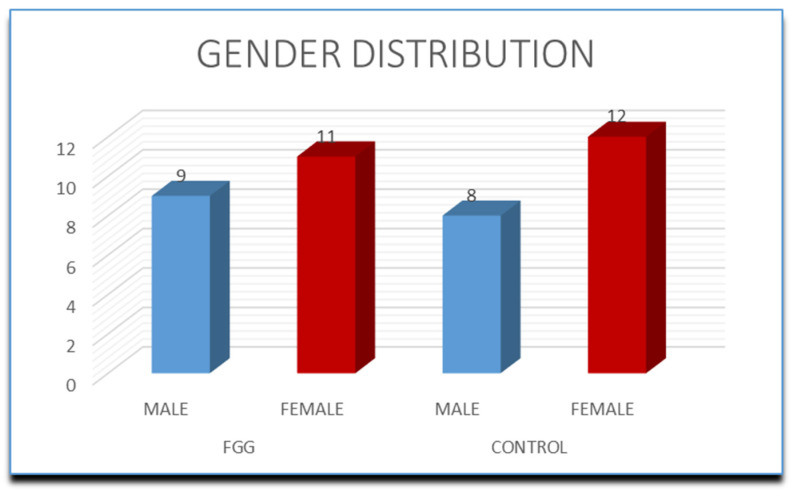
Gender distribution in the groups. FGG—free gingival graft.

The SS group included 20 participants, aged 30–61 (mean age 47.75 ± 10.97 years), of whom 9 (45%) were male and 11 (55%) were female. The gender difference was statistically insignificant (χ^2^ = 0.20, *p* = 0.655). Twelve of the participants in the control group were male (60%) and eight were female (40%) (χ^2^ = 0.80, *p* = 0.371). Patients’ age was between 31 and 65 years (mean = 51.10 ± 11.70). The gender distribution is presented in Figure 1.

### 2.4. Radiological Examination

**Figure 2:**
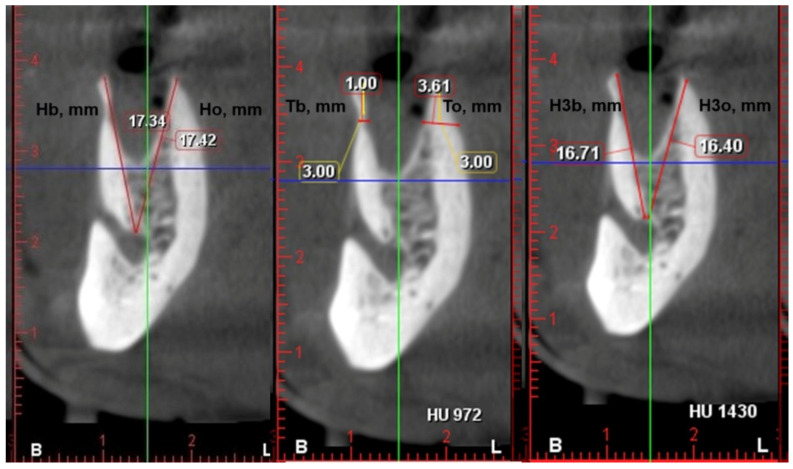
Measurement of the height and thickness of the socket plates using cone-beam computed tomography. Hb—buccal plate height on day 1; Ho—oral plate height on day 1; Tb—buccal plate thickness on day 1; To—oral plate thickness on day 1; H3b—buccal plate height 3 months later; H3o—oral plate height 3 months later.

Cone-beam computed tomography (CBCT) is an essential tool for three-dimensional diagnostics of the alveolar ridge [16,17]. CBCT images were used for the postoperative measurements. They were performed immediately after the extraction and 3 months later. The measurements were taken on a paraxial section in the middle of the socket immediately after the extraction (Hb—buccal plate height; Ho—oral plate height; Tb—buccal plate thickness; To—oral plate thickness) and after 3 months (H3b—buccal plate height after 3 months; H3o—oral plate height after 3 months). The plate heights were measured from the uppermost point of the wall to the uppermost point of the maxillary sinus/mandibular canal. Plate thickness was measured 3 mm below the highest point of the wall. When the inter-radicular septa at the molar post-extraction sockets were preserved, measurements were taken in the middle of the mesial and distal parts of the socket. Therefore, the number of measurements was bigger than the number of participants (Figure 2).

### 2.5. Surgical Intervention

**Figure 3:**
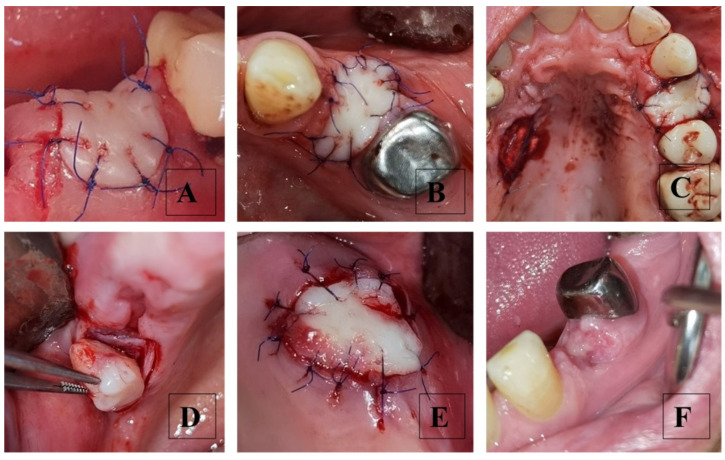
Socket sealing with free gingival grafts. (**A**–**C**)—palatal grafts in their recipient sites; (**D**)—tuberosity graft harvesting; (**E**)—tuberosity graft in the recipient site; (**F**)—graft healing—day 12.

All surgical interventions were performed by the principal investigator (R.Y.). Tooth extraction was performed after local anesthesia with 4% Articaine. Teeth with multiple roots were separated and extracted one by one to avoid bone fractures. Socket debridement and rinsing were performed. In the control group, the soft tissue margins were sutured with 5/0 polyamide. In the SS group, the socket orifice was prepared as a recipient site by de-epithelization of the marginal gingiva and hemostasis. An FGG was harvested from the hard palate or maxillary tuberosity under local anesthesia using a sterile foil as a template to repeat the shape and size of the recipient site (the graft size can slightly exceed the size of the socket orifice). Palatal grafts were harvested from the premolar–molar area but at least 2 mm below the gingival margin and without passing the distal edge of the first molar. Tuberosity grafts are usually thicker and can be used when the wisdom tooth is missing and there are enough tissues. The grafts must be transferred as soon as possible to the recipient site and fixed with single interrupted sutures using a 6/0 non-resorbable monofilament polyamide suture (Figure 3).

Antibiotics (amoxicillin with clavulanic acid or azithromycin), analgesics (nimesulide 100 mg twice daily or ibuprofen 400 mg twice daily for 3 days and then if needed), probiotics, and mouth rinse (0.12% chlorhexidine—Eluperio^®^, Pierre Fabre, France) were prescribed. Postoperative instructions for oral hygiene and diet were given, and suture removal was scheduled for after 7–10 days.

### 2.6. Statistical Analysis

Descriptive statistics measures (mean and standard deviation (SD), median, and interquartile range (IQR)) were used for data analysis and results presentation using IBM SPSS v.25.0, MS Office Excel, and Jamovi Statistical Software Version 2.3.28. Hypothesis testing was performed using parametric (*t*-test, Pearson correlation) and non-parametric tests (the Mann–Whitney U test, Pearson’s chi-squared test, Spearman correlation). Statistically significant values (*p* < 0.05) were presented and further analyzed.

## 3. Results

**Figure 4:**
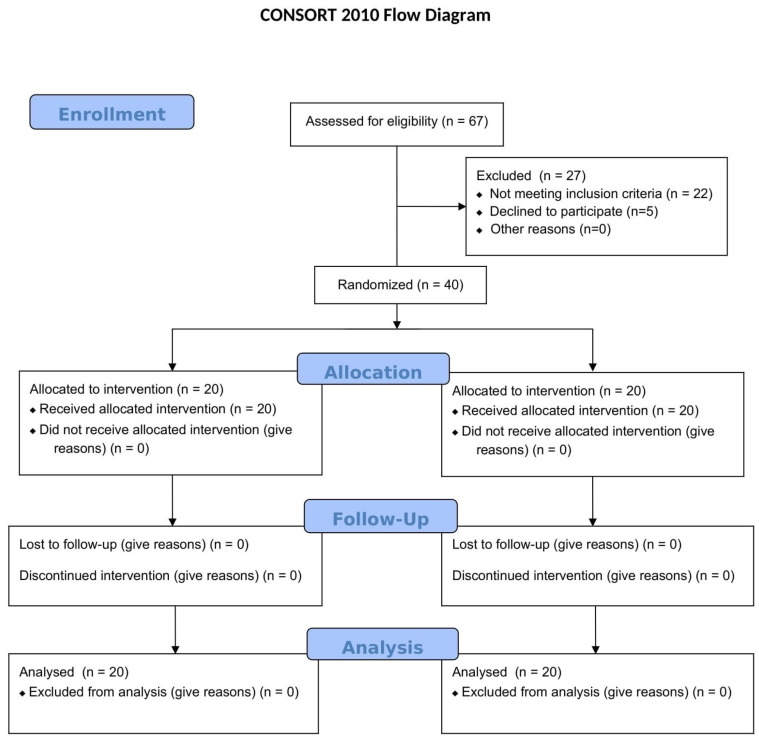
Flow diagram of the study subjects.

Figure 4 displays the flow diagram of the participants.

Table 1 displays the measured values in the SS and control groups.

Vertical bone resorption after 3 months was significant in both groups: 1.65 ± 0.92 mm in the buccal wall and 1.27 ± 0.56 mm in the oral wall in the SS group, and 3.47 ± 1.96 mm in the buccal wall and 2.08 ± 1.10 mm in the oral wall in the control group (Table 2).

**Figure 5:**
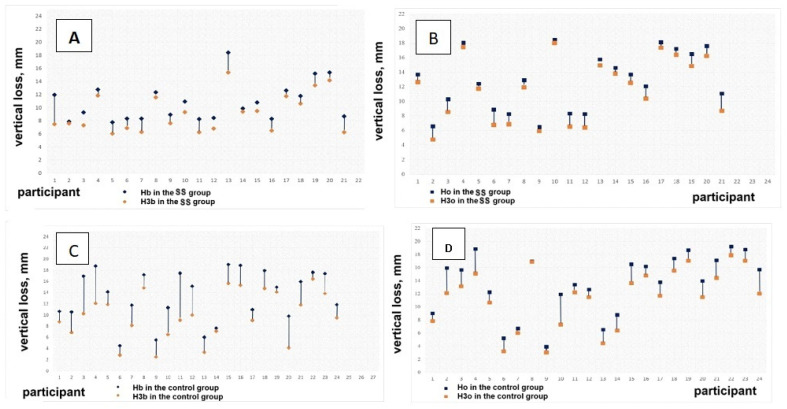
Vertical resorption of the plates in millimeters: (**A**,**B**)—in the SS group; (**C**,**D**)—in the control group.

Figure 5 presents the vertical resorption of the socket walls in the SS and control groups. The diagrams show more pronounced resorption in the group without socket sealing.

The difference between the vertical resorption of the buccal and oral walls in the SS group was statistically insignificant.

The difference (0.38 mm) between the vertical loss of the two plates in the experimental group was statistically insignificant (Mann–Whitney = 167.500, *p* = 0.182). The correlation between their resorption was weak and statistically insignificant (Spearman’s r = 0.29, *p* = 0.207).

A statistically significant difference (1.39 mm) was found between the vertical resorption of the buccal and oral walls in the control group (t = 3.01, *p* = 0.005). The correlation between the resorption of the two walls was weak and statistically insignificant (Pearson’s ρ = 0.21, *p* = 0.333).

**Figure 6:**
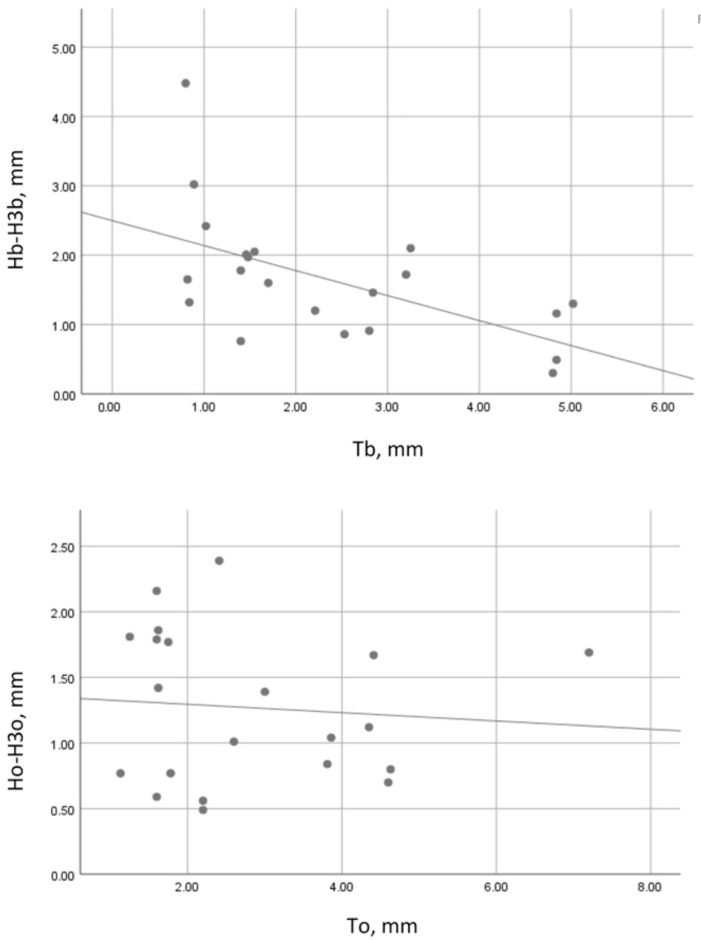
Correlation between wall thickness and vertical resorption in the SS group. Tb—buccal plate thickness on day 1; Hb-H3b—vertical resorption of the buccal socket wall; To—oral plate thickness on day 1; Ho-H3o—vertical resorption of the oral socket wall.

In the SS group, the correlation between the wall thickness and its vertical resorption was strong, negative, and statistically significant (Spearman’s r = −0.543, *p* = 0.011) at the buccal wall and weak, negative, and statistically insignificant (Pearson’s r = −0.086, *p* = 710) at the oral socket wall (Figure 6).

**Figure 7:**
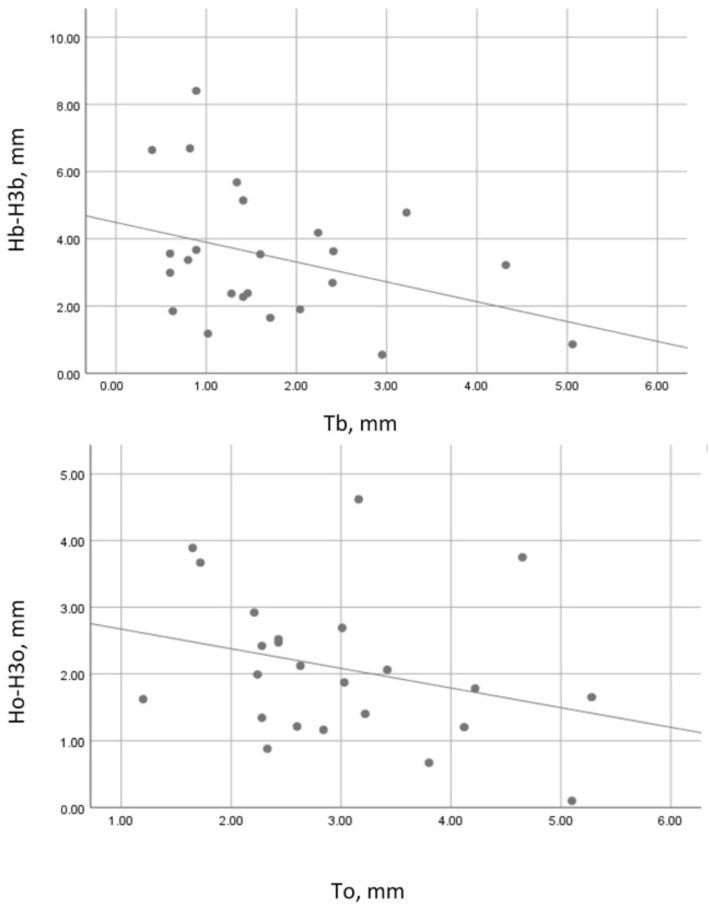
Correlation between wall thickness and vertical resorption in the control group. Tb—buccal plate thickness on day 1; Hb-H3b—vertical resorption of the buccal socket wall; To—oral plate thickness on day 1; Ho-H3o—vertical resorption of the oral socket wall.

In the control group, the correlation between the wall thickness and its vertical loss was moderate, negative, and statistically insignificant (Pearson’s r = −0.358, *p* = 0.086) at the buccal socket wall and weak, negative, and statistically insignificant (Pearson’s r = −0.287, *p* = 0.175) at the oral socket wall (Figure 7).

**Figure 8:**
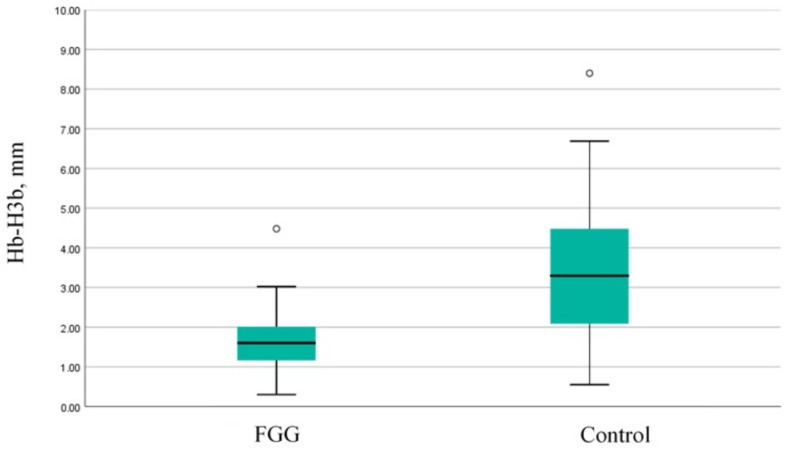
Comparison of the vertical resorption of the buccal socket wall between the groups. FGG—free gingival graft; Hb-H3b—vertical resorption of the buccal socket wall.

The vertical loss of the buccal walls was more pronounced in the control group compared to the SS group (mean difference—1.82 mm; t =−4.07, *p* < 0.0001) (Figure 8). This was valid for both thick (>2 mm) (t =−2.79, *p* = 0.023) and thin buccal walls (≤2 mm) (Mann–Whitney = 137.500, *p* = 0.013).

**Figure 9:**
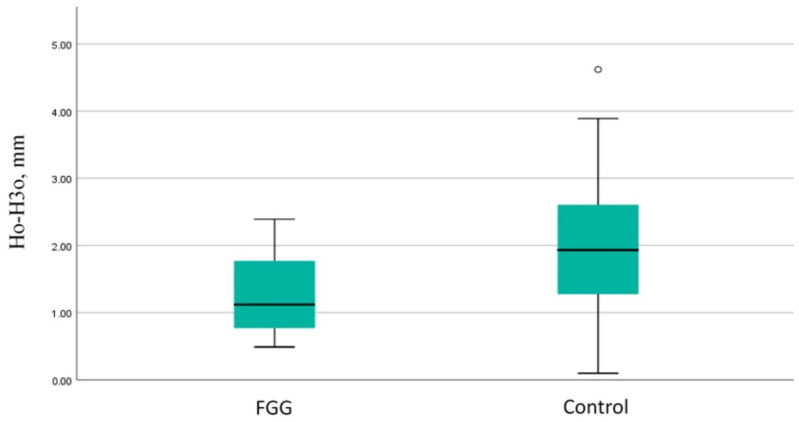
Comparison of the vertical resorption of the oral socket wall between the groups. FGG—free gingival graft; Ho-H3o—vertical resorption of the oral socket wall.

Similarly, the vertical loss of the oral walls was more pronounced in the control group (mean difference—0.81 mm; t = −3.17, *p* = 0.003) (Figure 9).

## 4. Discussion

Tooth extraction in the esthetic area requires hard and soft tissue preservation for the subsequent implant treatment. Various methods for soft tissue management at post-extraction sites have been proposed, such as pedicle flaps, coronary displaced flaps, and the modified tunnel technique [18]. A major disadvantage of these methods is the flap preparation, which results in additional tissue loss due to surgical trauma and the exposure of the thin buccal plate. Furthermore, the separation of the periosteum from the bone leads to resorption of the latter. Other complications such as soft tissue retraction and scarring have also been observed [19]. Various socket preservation methods have been recently adopted to avoid these limitations, such as socket sealing with autogenous soft tissue grafts [20].

Graft survival depends on its supply from the underlying blood coagulum and the surrounding soft tissues. Therefore, it is considered that better graft nutrition is achieved in sockets that are not filled with bone substitutes. On the other hand, the socket can be filled with autologous platelet concentrates (APCs), such as platelet-rich plasma and platelet-rich fibrin, which release an abundance of growth factors and other bioactive molecules [21,22]. The positive effect of APCs on bone and soft tissue healing has been well documented in the literature [23,24,25].

The SS and control groups in our study demonstrated significant vertical resorption over 3 months.

In the SS group, the resorption of the buccal socket wall was 1.65 ± 0.92 mm, and the resorption of the oral wall was 1.27 ± 0.57 mm. The mean difference between them was 0.38 mm, which suggested that the resorption was similar for both walls. The correlation between them was weak, negative, and statistically insignificant, i.e., both walls did not affect each other. The correlation between the wall thickness and its vertical resorption over 3 months was strong, negative, and statistically significant at the buccal wall and weak, negative, and statistically insignificant at the oral wall. It can be concluded that buccal resorption depended on the thickness of the wall.

In the control group, the resorption of the buccal socket wall was 3.47 ± 1.96 mm, and the resorption of the oral wall was 2.08 ± 1.10 mm. The mean difference between them was 1.39 mm, which suggested that the resorption of the two walls was uneven. The correlation between the resorption of the walls was weak and statistically insignificant, e.g., both walls did not affect each other. Notably, the mean difference between the buccal and oral wall thicknesses was 1.26 mm. Moreover, the mean buccal wall thickness was ≤2 mm, while the oral wall thickness was >2 mm. Buccal walls with thicknesses of <2 mm have been associated with more evident bone resorption [26]. However, the thickness threshold below which the resorption accelerates has not been established yet. On the other hand, the correlation between the wall thickness and its vertical resorption over 3 months in the group was weak, negative, and statistically insignificant for both walls. Therefore, the socket wall thickness was not the only determining factor for the pronounced resorption.

Some authors consider a “critical value” in the wall thickness the threshold of 2 mm [27,28]. Others claim that this value is 1 mm, below which two or three times greater vertical resorption can be expected [29,30]. This can be attributed to the alveolar bone proper—a thin bone structure that covers the socket inside and disappears after tooth extraction.

As already described, the vertical resorption of the buccal wall was greater in the control group for both thin (≤2 mm) and thick walls (>2 mm). Accordingly, the determining factor for pronounced resorption in the group was the lack of SS. In addition, SS has a pivotal role in reducing the vertical resorption in sockets with thin and thick walls. Moreover, measuring the buccal wall thickness before extraction could serve as a prognostic marker for the amount of expected bone resorption [31]. This trial demonstrates that SS using free gingival grafts is a successful method for reducing post-extraction bone resorption. Furthermore, unlike most socket preservation methods, this technique does not involve flap reflection, which is another advantage since the denudation of the alveolar ridge has been associated with interruption of the blood supply, leading to external bone resorption [32].

### 4.1. Study Limitations

A major limitation of this study is the short follow-up period (3 months), which is informative for early implant placement but should be extended for delayed and late implantation. In addition, the sample size is relatively small, which leads to limited generalizability. A larger sample size with an expanded age range would provide more accurate results and allow for intergroup and intragroup analyses. Moreover, the correlation between the buccal wall width and vertical resorption was assessed by using only the threshold of 2 mm instead of using both 1 and 2 mm thresholds. This can be explained by the limited cases with buccal wall width ≤1 mm.

### 4.2. Future Directions

Further long-term randomized controlled clinical trials with larger sample sizes and greater age ranges are necessary to evaluate the use of FGGs for SS and compare the method to other socket sealing techniques. Study bias can be avoided using computer-generated randomization tools and software. This study supports the evidence that the buccal plate width influences its vertical resorption. However, future research should clarify if there is a critical threshold in the width and the exact correlation between the width and the subsequent vertical plate resorption.

## 5. Conclusions

Socket sealing with an FGG is a valuable method that eliminates the need for flap reflection and compensates for the soft tissue deficit when immediate implant placement or bone augmentation is required. In this research, the suggested method successfully reduced the vertical resorption of the post-extraction socket. Other important findings were that the determining factor for vertical resorption was the management of the post-extraction socket (SS versus secondary healing). This socket sealing method can significantly compensate for the resorption of the thin walls but cannot completely neutralize the wall thickness as a factor. Future long-term randomized clinical trials with a larger sample size should evaluate and compare FGGs and other socket-sealing methods, as well as their application with different socket-filling materials.

## Figures and Tables

**Table 1:** Measurements of height and thickness in the groups.

Group	Parameter/Unit	*N*	Mean	SD	Median	IQR	Range	Min	Max
SS	Hb/mm	21 *	10.77	2.91	9.88	4.15	10.66	7.76	18.42
	Tb/mm		2.37	1.46	1.70	2.02	4.22	0.80	5.02
	Ho/mm		12.82	4.02	12.91	8.24	12.00	6.48	18.48
	To/mm		2.82	1.56	2.20	2.50	6.07	1.13	7.20
	H3b/mm		9.14	2.90	7.62	5.01	9.36	6.04	15.40
	H3o/mm		11.55	4.35	11.90	8.79	13.23	4.76	17.99
Control	Hb/mm	24 *	13.40	4.54	14.54	6.91	14.51	4.49	19.00
	Tb/mm		13.54	4.63	14.80	7.34	15.35	3.88	19.23
	Ho/mm		1.73	1.19	1.41	1.52	4.66	0.40	5.06
	To/mm		2.99	1.07	2.74	1.43	4.08	1.20	5.28
	H3b/mm		9.94	4.24	9.73	7.12	13.92	2.51	16.43
	H3o/mm		11.46	4.51	12.05	7.61	14.89	3.00	17.89

SS—socket sealing; Hb—buccal plate height on day 1; Ho—oral plate height on day 1; Tb—buccal plate thickness on day 1; To—oral plate thickness on day 1; H3b—buccal plate height 3 months later; H3o—oral plate height 3 months later. * Number of measurements. It differs from the number of extracted teeth because preserved inter-radicular septa divided the socket into two sites, each measured individually.

**Table 2:** Vertical bone loss in the SS and control groups.

Group	Parameter/Unit	*N*	Mean	SD	Mean Diff.	95% CI		Test	*p*
						Lower	Upper		
SS	Hb	21	10.77	2.91	1.65	1.23	2.06	0.0000 *	<0.0001
	H3b		9.14	2.90					
	Ho		12.82	4.02	1.27	1.01	1.53	10.18 **	<0.0001
	H3o		11.55	4.35					
Control	Hb	24	13.40	4.54	3.47	2.64	4.29	8.66 **	<0.0001
	H3b		9.94	4.24					
	Ho		13.54	4.63	2.08	1.62	2.55	9.27 **	<0.0001
	H3o		11.46	4.51					

* Wilcoxon ranking test. ** Student’s *t*-test.

## Data Availability

The data presented in this study are available on request from the corresponding author due to privacy and ethical reasons.
